# Types and effects of feedback for emergency ambulance staff: a systematic mixed studies review and meta-analysis

**DOI:** 10.1136/bmjqs-2022-015634

**Published:** 2023-04-07

**Authors:** Caitlin Wilson, Gillian Janes, Rebecca Lawton, Jonathan Benn

**Affiliations:** 1 School of Psychology, University of Leeds, Leeds, UK; 2 Research and Development Department, Yorkshire Ambulance Service NHS Trust, Wakefield, UK; 3 Yorkshire Quality and Safety Research Group, Bradford Institute for Health Research, Bradford, UK; 4 Department of Nursing, Manchester Metropolitan University, Manchester, UK

**Keywords:** Prehospital care, Audit and feedback, Health services research, Decision making

## Abstract

**Background:**

Extensive research has been conducted into the effects of feedback interventions within many areas of healthcare, but prehospital emergency care has been relatively neglected. Exploratory work suggests that enhancing feedback and follow-up to emergency medical service (EMS) staff might provide staff with closure and improve clinical performance. Our aim was to summarise the literature on the types of feedback received by EMS professionals and its effects on the quality and safety of patient care, staff well-being and professional development.

**Methods:**

A systematic review and meta-analysis, including primary research studies of any method published in peer-reviewed journals. Studies were included if they contained information on systematic feedback to emergency ambulance staff regarding their performance. Databases searched from inception were MEDLINE, Embase, AMED, PsycINFO, HMIC, CINAHL and Web of Science, with searches last updated on 2 August 2022. Study quality was appraised using the Mixed Methods Appraisal Tool. Data analysis followed a convergent integrated design involving simultaneous narrative synthesis and random effects multilevel meta-analyses.

**Results:**

The search strategy yielded 3183 articles, with 48 studies meeting inclusion criteria after title/abstract screening and full-text review. Interventions were categorised as audit and feedback (n=31), peer-to-peer feedback (n=3), postevent debriefing (n=2), incident-prompted feedback (n=1), patient outcome feedback (n=1) or a combination thereof (n=4). Feedback was found to have a moderate positive effect on quality of care and professional development with a pooled effect of d=0.50 (95% CI 0.34, 0.67). Feedback to EMS professionals had large effects in improving documentation (d=0.73 (0.00, 1.45)) and protocol adherence (d=0.68 (0.12, 1.24)), as well as small effects in enhancing cardiac arrest performance (d=0.46 (0.06, 0.86)), clinical decision-making (d=0.47 (0.23, 0.72)), ambulance times (d=0.43 (0.12, 0.74)) and survival rates (d=0.22 (0.11, 0.33)). The between-study heterogeneity variance was estimated at σ^2^=0.32 (95% CI 0.22, 0.50), with an I^2^ value of 99% (95% CI 98%, 99%), indicating substantial statistical heterogeneity.

**Conclusion:**

This review demonstrated that the evidence base currently does not support a clear single point estimate of the pooled effect of feedback to EMS staff as a single intervention type due to study heterogeneity. Further research is needed to provide guidance and frameworks supporting better design and evaluation of feedback interventions within EMS.

**PROSPERO registration number:**

CRD42020162600.

**WHAT IS ALREADY KNOWN ON THIS TOPIC**

- Within the wider healthcare context, interventions based on audit and feedback are well researched, with systematic reviews suggesting that feedback results in small to moderate improvements to patient care through enhancing clinical performance. Existing reviews have not included studies within prehospital emergency care, despite a growing body of research suggesting that feedback to emergency medical service (EMS) personnel could improve staff well-being and patient safety.

**WHAT THIS STUDY ADDS**

- This review summarises the literature on the types of feedback received by EMS personnel and its effects on quality of care and professional development.

**HOW THIS STUDY MIGHT AFFECT RESEARCH, PRACTICE OR POLICY**

- The review findings suggest that feedback to EMS professionals may improve performance and patient care; and highlight the need for more sophisticated feedback designs and robust evaluations within EMS.

## Introduction

The phenomenon of feedback is well researched within healthcare, including, for example, debriefing, patient experience feedback and feedback from incident reporting.^1–3^ The feedback type investigated most frequently in clinical settings is audit and feedback, also known as clinical performance feedback, which is defined as: ‘any summary of clinical performance over a specified time period’.^4^ A Cochrane review published in 2012 suggested that audit and feedback results in small to moderate improvements in patient care by enhancing healthcare professionals’ compliance with desired clinical practice.^5^

Patient outcome feedback is also increasingly used to support reflection and self-evaluation of clinical performance.^6^ This information enables clinicians to gain insight and knowledge, and may improve diagnostic ability and prompt other behaviour changes.^7^ It may also improve calibration, that is, the relationship between a clinician’s confidence in their diagnostic accuracy and the true accuracy of the diagnosis, thereby improving patient care and patient safety.^6–8^

Feedback and follow-up on patients and performance is particularly difficult for emergency medical service (EMS) personnel. Although their work involves engaging and communicating with other parts of the health service during handover and referral of patients, EMS operate largely in silos with a geographically dispersed workforce delivering discrete episodes of care without direct supervision.^9^ This disconnect from the subsequent care team and episodic nature of autonomous EMS work means that EMS staff struggle to obtain feedback.

Extensive research has been conducted into the effects of feedback interventions within many healthcare settings; however, in 2012 relatively little work had been undertaken within EMS, as illustrated by the lack of EMS studies eligible for inclusion in the Cochrane review of audit and feedback.^5^ Since then, a growing body of literature has been published on feedback within EMS suggesting that enhancing feedback and follow-up for EMS staff might improve clinical performance and staff well-being,^10–12^ but this has not yet been synthesised comprehensively.

Our aim was to summarise the literature on the types of feedback received by EMS professionals and their effects on quality and safety of patient care. The following questions were addressed:

- What are the intervention types, design elements, potential mechanisms and reported effects of feedback interventions to EMS professionals?
- How do EMS professionals perceive current feedback provision?
- What are the reported contextual factors (eg, barriers, facilitators, opportunities) for effective feedback to EMS staff?

## Methods

The study was defined as a ‘systematic mixed studies review’, as preliminary searches identified important quantitative, qualitative and mixed-methods studies of relevance to the review aims.^13^ The review was registered on PROSPERO (CRD42020162600) and the protocol published a priori.^14^

### Search strategy

The search strategy was developed in collaboration with an ambulance service librarian and included three facets: ambulance staff, feedback and feedback content (online supplemental appendix 1). Ovid (MEDLINE, Embase, AMED, PsycINFO, HMIC), EBSCO (CINAHL) and Web of Science were searched from their respective inception dates and searches last updated on 2 August 2022.

10.1136/bmjqs-2022-015634.supp1 Supplementary data

### Study inclusion and exclusion criteria

English language primary studies of qualitative, quantitative and mixed-methods methodology were included if they described, evaluated or discussed feedback to EMS professionals. Reasons for excluding non-English studies were pragmatic (ie, lack of funding for translation, reducing the number of retrieved articles and therefore screening time) and theoretical (ie, the review targeted the Anglo-American EMS system involving paramedics rather than physicians). Morrison *et al* 15 and Moher *et al* 16 provided reassurance that this exclusion would not introduce bias in systematic reviews within medicine and healthcare.

Feedback was defined as the systematic provision of information to EMS professionals regarding their performance using process metrics, patient outcome metrics or both types. Grey literature was excluded along with articles focused on automated feedback by devices (eg, automated external defibrillators) and feedback based on hypothetical data as these were considered distinct cases of feedback with their own existing literature syntheses.^17–25^ Educational settings were excluded as the review focused on real-world performance.

### Study selection

CW independently screened the full set of titles and abstracts retrieved in two passes: (1) setting (prehospital emergency care) and (2) intervention (feedback to EMS professionals). Emily Parker independently screened a random 10% subset and disagreements were resolved through discussion. Although independent review of all search results by two reviewers is recommended to reduce the probability of missing relevant studies, dual screening a small percentage of records is an acceptable alternative that pragmatically balances thoroughness and resource use.^26 27^ Cohen’s kappa was calculated to determine the level of agreement between reviewers for screening (strong agreement for setting k_S_=0.85, moderate agreement for intervention k_I_=0.78). CW assessed eligibility of articles selected for full-text review with JB verifying articles excluded at this stage.

### Study quality assessment

CW appraised the methodological quality of individual studies using the Mixed Methods Appraisal Tool.^28^ This tool allowed for the appraisal of qualitative research, randomised controlled trials, non-randomised studies, quantitative descriptive studies and mixed-methods studies within a single framework. JB confirmed the ratings assigned to each study and disagreements were resolved through discussion.

### Data extraction

CW performed data extraction using a comprehensive, standardised extraction template that was tested and iterated by the review team (online supplemental appendix 2). A second researcher (GJ, RL or JB) checked the data extraction forms for accuracy and detail for a total of 20% (n=10) of studies. CW contacted four authors for missing data to enable inclusion in the meta-analysis, with one providing additional data, two unable to provide data due to study methods or governance issues and one not responding. We included the study where additional data were provided in the meta-analysis, while effect sizes of the remaining three studies were reported descriptively.

### Data synthesis

Studies were divided into three a priori defined categories:

1. Empirical studies reporting feedback interventions within EMS, subcategorised into evaluative studies and descriptive case studies.
2. Empirical non-interventional studies of feedback within EMS.
3. Empirical studies in which feedback to EMS was one, but not the sole or primary focus of the study—these studies were not included in the analysis but are introduced in the discussion to highlight opportunities for enhanced feedback interventions.

The resulting groupings contained quantitative, qualitative and mixed-methods studies. As the review questions could be addressed by both qualitative and quantitative data, a convergent integrated approach was chosen, which involved synthesis occurring simultaneously (‘*convergent’*) and data being transformed to combine quantitative and qualitative data (‘*integrated’*).^13 29 30^ Specifically, we transformed quantitative data into qualitative data by converting it into textual descriptions to be included in our narrative synthesis.^29^ An example of this would be 41.7% of respondents in the Cash *et al* 31 survey reporting to have received patient outcome feedback, compared with 54.7% receiving medical care feedback, which we converted to ‘patient outcome feedback was less frequent than clinical performance feedback’.

We performed a narrative synthesis for data relating to feedback types and design elements, EMS professionals’ perceptions of feedback provision and key contextual factors. In line with guidance for narrative synthesis^32^ and best practices within the broader field of implementation science, we used theory to further understand the mechanisms of effective feedback.^33 34^ To describe feedback design elements we used clinical performance feedback intervention theory (CP-FIT), which is a prominent and widely adopted theory within the implementation science literature based on a meta-synthesis of 73 feedback interventions.^35^ Our own qualitative work on feedback to emergency ambulance staff found CP-FIT to have good face validity when exploring causal mechanisms at an abstract level, for example, ‘processing and reflection’.^12^

To identify potential mechanisms at a more detailed level for this review, we used behaviour change theory, which aims to identify active ingredients of interventions seeking to change behaviour^36^ and has previously been used to synthesise evidence from audit and feedback interventions.^37 38^ CW identified potential causal mechanisms deductively using an established list of mechanisms of action from behaviour change theory,^36^ with a researcher experienced in behaviour change theory coding (RL) verifying the assigned codes of a 20% (n=10) random sample. Disagreements were resolved through discussion. Once agreement was reached, CW reviewed the remaining studies.

Lastly, we analysed feedback effects by conducting post hoc random-effects multilevel meta-analyses of quantitative evaluative studies, including tabulation and aggregation of standardised mean differences (Cohen’s *d*) and corresponding 95% CIs using the package ‘metafor’ in R V.4.1.3.^39^ Cohen’s *d* is an effect size that evaluates the difference between two means and is interpreted as small (d=0.2), medium (d=0.5) or large (d=0.8).^40^ Cohen’s *d* is particularly useful in meta-analyses of studies that assess the same (continuous) outcome but measure it using different scales, as it allows each study result to be standardised to a uniform scale and therefore allows studies to be combined.^27^ Effect sizes were extracted directly, approximated from median and IQRs or calculated from raw data.^27 41–44^ Individual meta-analyses were conducted for each outcome category, as well as a combined meta-analysis to quantify the effects of feedback overall on EMS care quality and safety.^45^ A multilevel approach was chosen for the overall meta-analysis to account for including multiple effect sizes from individual studies.^46^ Where studies reported multiple effect sizes within a single outcome category, the largest effect size was chosen, in line with a ‘proof of concept’ approach.^47^ Relationships in the data were meta-analysed using subgroup analyses informed by CP-FIT’s desirable feedback intervention design elements, feedback type and study quality.^35^

Between-study heterogeneity was assessed using χ^2^ test, I^2^, τ^2^ and prediction intervals (PIs), following guidance to use multiple measures to characterise statistical heterogeneity in meta-analyses due to the relative strengths and weaknesses of the different measures available.^27 48^ The χ^2^ test (Cochran’s Q) is the traditional test to detect whether heterogeneity is present in meta-analyses and examines the null hypothesis that all studies are evaluating the same effect.^49^ Cochran’s Q has low power to detect differences with small numbers of studies and high power when there are many studies, so alpha was set to p<0.10 for the individual meta-analyses of each outcome category and p<0.05 for the overall meta-analysis.^27^ To describe the extent of between-study variability due to heterogeneity, the I^2^ statistic was calculated and interpreted as: not important (0%–40%), moderate (30%–60%), substantial (50%–90%) and considerable (75%–100%).^27^ I^2^ can be biased in small and large meta-analyses,^50 51^ so τ^2^ was used to quantify the variance of the true effect sizes underlying our data in a way that was insensitive to the number of studies.^48^ Due to our overall meta-analysis being a multilevel analysis, between-study heterogeneity variance is denoted as σ^2^ instead of τ^2^.^48^ As τ^2^ and σ^2^ are difficult to interpret, we used their results to calculate 95% PIs, which provide an estimate of the expected range of true effects in future studies using the same scale as the effect size metric (ie, Cohen’s *d*).^27 52^

### Quality of evidence

As this review’s convergent synthesis design required data transformation and integration, the certainty associated with the whole body of literature was not assessed and instead individual study quality reported. This aligns with recent guidance, warning against using Grading of Recommendations Assessment, Development and Evaluation (GRADE)/Confidence in Evidence from Reviews of Qualitative research (CERQual) in mixed-studies systematic reviews.^29^

## Results

**Figure 1:**
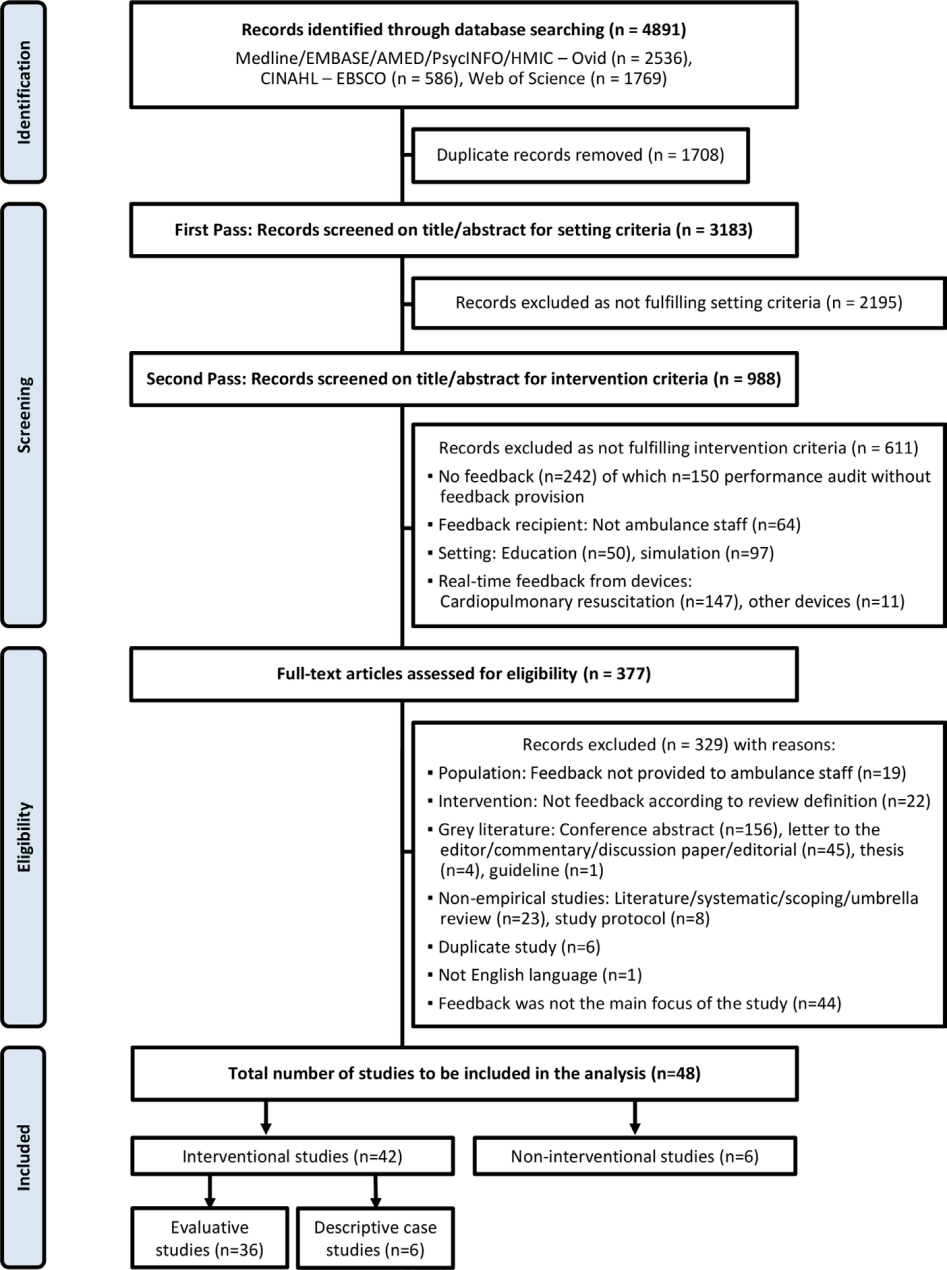
Preferred Reporting Items for Systematic Reviews and Meta-Analyses (PRISMA) flow diagram.

Our cumulative search identified 4891 results (figure 1). Once duplicates were removed, 3183 titles and abstracts were screened for inclusion. Of these, 2195 were excluded based on the setting and 611 based on the intervention criteria. The remaining 377 articles underwent full-text review, resulting in 329 exclusions and a total of 48 articles included in the analysis.

### Study characteristics

An overview of included studies is presented in table 1 with a detailed breakdown in online supplemental appendix 3. There were 42 interventional studies and six non-interventional studies. Studies were published between 1989 and 2022 with 73% (n=35) published in the last 10 years, and originated most frequently in the USA (n=19, 40%). The most common study design was cross-sectional (n=30, 63%). Most studies were situated within paramedic emergency services (n=39, 81%), but emergency operations centres (n=6, 13%) were also represented.

**Table 1:** Characteristics of included studies

Characteristic	Interventional feedback studies (n=42)		Non-interventional feedback studies (n=6)		Total (N=48)	
	n	%*	n	%*	n	%*
Year						
1989–1999	6	14	1	17	7	15
2000–2009	3	7	–		3	6
2010–2019	26	62	2	33	28	58
2020–2022	7	17	3	50	10	21
Country						
USA	16	38	3	60	19	40
Germany	6	14	–	–	6	13
UK	4	10	2	33	6	13
Sweden	3	7	–	–	3	6
Netherlands	2	5	–	–	2	4
Australia	2	5	–	–	2	4
Other or multiple countries	9	21	1	17	10	21
Study design						
Cohort study	4	10	–	–	4	8
Case–control study	1	2	–	–	1	2
Cross-sectional study	30	71	–	–	30	63
Quantitative descriptive study	4	10	3	50	7	15
Qualitative study	1	2	3	50	4	8
Mixed-methods study	2	5	–	–	2	4
Context						
Paramedic emergency services	33	79	6	100	39	81
Emergency operations centre	6	14	–	–	6	13
Ambulance service organisation	2	5	–	–	2	4
Other or multiple contexts	1	2	–	–	1	2

*Sections of individual columns may not add up to exactly 100% due to rounding.

### Quality assessment

All studies were assessed as having clear research questions and collected data addressing these (online supplemental appendices 3 and 4). The qualitative and mixed-methods studies were of high quality. Several quantitative descriptive studies (n=5, 10%) omitted reporting on non-response bias, and were therefore of high-to-moderate quality, with the remaining being of high quality. Many of the quantitative non-randomised studies were of moderate quality due to confounders not being accounted for in the study design and analysis (n=23, 48%), as well as not reporting on representativeness of participants (n=6, 13%) and intervention fidelity (n=30, 63%).

### Feedback intervention types and designs within EMS (interventional studies)

The interventional studies (n=42) described 37 unique EMS feedback interventions. Sources of feedback included researchers (n=17, 40%), ambulance service managers (n=14, 33%), peers (n=6, 14%) and external databases (n=1, 2%). Feedback was provided to organisations (n=8, 19%), ambulance crews/teams (n=16, 38%), individual clinicians (n=10, 24%) or a combination (n=7, 17%). An example of an organisational-level intervention provided ambulance services with feedback on their care bundle performance for myocardial infarction and stroke via monthly teleconferences and weekly control charts of performance data.^53^ Weston *et al*, on the other hand, provided individual paramedics with a single page of written feedback on their performance in cardiac arrests, including chest compression depth and rate.^54^

Feedback was delivered as a stand-alone initiative (n=17, 40%), or as part of wider organisational (n=12, 29%) or educational (n=13, 31%) interventions. An example of a stand-alone initiative was the provision of hospital-directed feedback to an EMS organisation on compliance with state protocols and documentation.^55^ Other studies described feedback being implemented alongside training^56^ or new equipment and guidelines.^57^

The clinical topic was varied with the most frequent being cardiac arrest (n=13, 31%), myocardial infarction (n=10, 24%), stroke (n=3, 7%) and trauma (n=2, 5%). Other studies included multiple conditions or all patients (n=12, 29%), with the remaining focusing on paediatrics (n=1, 2%) and non-conveyed patients (n=1, 2%). Overall, interventional studies described six different feedback types according to the typology developed in our previously published exploratory interview study^12^: audit and feedback (n=31, 74%), postevent debriefing (n=2, 5%), peer-to-peer feedback (n=3, 7%), incident-prompted feedback (n=1, 2%), audit and patient outcome feedback (n=4, 10%) and patient outcome feedback (n=1, 2%) (table 2).

**Table 2:** Characteristics of feedback to EMS professionals identified in this systematic review

Feedback type	Feedback on professional practice	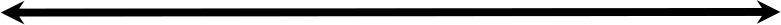				Patient outcome feedback
	Audit and feedback	Postevent debriefing	Peer-to-peer feedback	Incident-prompted feedback	Audit and patient outcome feedback	Patient outcome feedback
Illustrative example	Feedback to EMS staff and ambulance stations by the research team on adherence to corticosteroid administration^60^	Feedback to EMS staff in a group setting following a resuscitation attempt using data collected from audit forms and defibrillator downloads with input from other EMS staff and the clinical training team^127^	Feedback to an EMS professional in a group setting on an everyday professional situation described by the feedback recipient with other EMS professionals offering their reflections and ideas on how to best achieve efficient professional care^73^	Feedback to EMS staff and ambulance stations on the observed trends of reported patient safety incidents, including patient outcomes and clinical management^58^	Feedback to EMS staff on their performance of EMS stroke quality measures, ambulance times and the patients’ final diagnosis at hospital discharge^63^	Feedback to EMS dispatchers by EMS staff on scene regarding patients’ primary condition when the ambulance arrived on scene, non-transport by the ambulance and level of priority^59^
Feedback interventions reviewed, n (%)	31 (74)	2 (5)	3 (7)	1 (2)	4 (10)	1 (2)
Data	(Most commonly) Aggregated	Individual or aggregate	Individual	Individual	Individual or aggregate	Individual
Content	Clinical performance metrics and adherence to these	EMS treatment and management plan	The views of peers on the EMS treatment and management plan	Analysis of EMS patient safety incidents	Clinical performance metrics and outcomes of patients	Outcomes of patients (eg, diagnosis, hospital treatment, management plan)
Set-up	Formal	Formal	Formal or informal	Formal	Formal	Formal or informal
Comparison	Explicit benchmarks	May include explicit benchmarks	Indirect comparison with other clinicians	No explicit comparison	May include explicit benchmarks	No explicit comparison
(Anticipated) Effects	Change clinician behaviour: improve clinical performance	Change clinician behaviour: improve clinical performance, provide support	Change clinician behaviour: improve patient management skills, provide peer support	Change clinician behaviour: reduce patient safety incidents	Change clinician behaviour: improve clinical performance, improve clinicians’ diagnostic ability	Change clinician behaviour: Improve clinicians’ diagnostic ability and patient management skills

EMS, emergency medical service.

Feedback was provided on individual (n=18, 43%), aggregate (n=10, 24%) or individual and aggregate patient cases (n=6, 14%) and in all but one study consisted of ‘push’ model feedback, that is, not in response to recipients actively seeking feedback.^58^ Only six studies (14%) reported that recipients were offered a comparison or benchmark alongside the feedback. The format of feedback interventions included written (n=14, 33%), verbal (n=10, 24%) or a combination of strategies (n=5, 12%). Visual elements were seldom reported but included line charts, viewing recordings of resuscitation performance and green/red highlighting on written reports. Frequency varied from after each call to only once or every 6 months, with lag time similarly varying from 48 hours to 3 months.

Many of the design characteristics discussed in the audit and feedback literature, for example, timeliness, user-friendly designs and inclusion of patient-level data, were not explicitly considered and no studies compared different feedback types. No studies reported using existing feedback theory (eg, CP-FIT); although one paper referenced audit and feedback literature,^59^ five used quality improvement methods,^53 60–62^ and a further paper acknowledged the challenge of sustainability within implementation science.^63^

### Outcomes and effectiveness of feedback interventions (interventional studies)

The feedback interventions reported in the evaluative interventional studies (n=36) measured a number of quality and safety outcomes, including process metrics—such as ambulance times (n=13, 36%, eg, arrival times, on-scene times, call-to-needle times), protocol adherence (n=12, 12%), cardiac arrest performance (n=10, 28%), clinical decision-making including non-conveyance decisions (n=6, 17%) and documentation (n=5, 14%)—as well as patient outcomes such as survival rates (n=8, 22%) and patient satisfaction (n=1, 3%). The remaining descriptive case studies (n=6) simply demonstrated that feedback was possible in the prehospital emergency care setting, for example, Stella *et al*,^58^ who reported how incident reporting data could be used to provide feedback to EMS professionals.^58^

**Figure 2:**
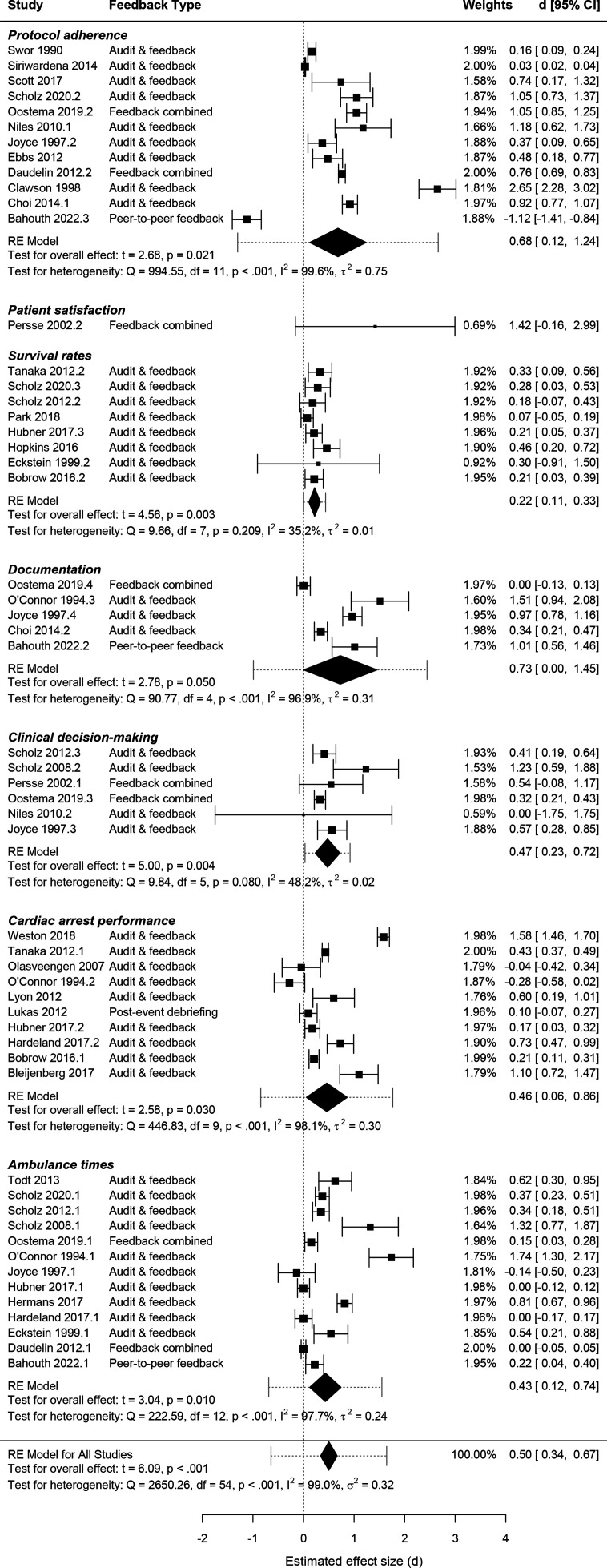
Forest plot of standardised mean differences and corresponding 95% CIs for feedback interventions in emergency medical service (EMS). RE, random effects.^128 129^

Of the 36 evaluative interventional studies, 30 (83%) provided sufficient data to calculate standardised effect sizes and were tabulated according to study outcomes (figure 2). All 30 studies were quantitative and non-randomised with the majority providing before-and-after comparison. Feedback to EMS professionals was found to have statistically significant positive effects in 73% (n=40) of the 55 extracted outcome measures, with the remaining outcome measures indicating non-significant effects in a positive direction (n=7, 13%), negative direction (n=4, 7%) or no effect (n=4, 7%). Outcome measures are listed in online supplemental appendix 3 and included, for example: stroke care bundle delivery (*protocol adherence*),^53^ satisfaction level of patients (*patient satisfaction*),^64^ survival to hospital discharge (*survival rates*),^65^ adequate documentation pertaining to physical examination (*documentation*),^66^ patients transported directly to the catheterisation laboratory (*clinical decision-making*),^67^ chest compressions per minute (*cardiac arrest performance*)^62^ and EMS run time (*ambulance times*).^68^

Overall, feedback had a moderate positive effect with a standardised mean difference for all outcome measures of d=0.50 (95% CI 0.34, 0.67). There was evidence of substantial between-study heterogeneity (I^2^=99%, 95% CI 98%, 99%) at a statistically significant level (Q=2650.26, p<0.001). Variance of between-study heterogeneity was estimated at σ^2^=0.32 (95% CI 0.22, 0.50), resulting in a wide PI (−0.64, 1.64), indicated in dotted lines on either side of the pooled effect size in figure 2. Our calculated PI illustrates the uncertainty in the pooled estimate for EMS feedback effects and crosses the line of no effect, so the true effect in future studies may be negative, null or higher than our pooled estimate.^52^

Separate meta-analyses for each outcome category revealed that feedback had a large effect in improving documentation (d=0.73 (0.00, 1.45)) and protocol adherence (d=0.68 (0.12, 1.24)), as well as small effects in enhancing cardiac arrest performance (d=0.46 (0.06, 0.86)), clinical decision-making (d=0.47 (0.23, 0.72)), ambulance times (d=0.43 (0.12, 0.74)) and survival rates (d=0.22 (0.11, 0.33)). Heterogeneity was substantial for all outcome categories except for survival rates (I^2^=35.2%) and clinical decision-making (I^2^=48.2%), which fell into the moderate range of the I^2^ index. Our results indicated moderate between-study heterogeneity at a statistically significant level for clinical decision-making (Q=9.84, p=0.080), but not for survival rates (Q=9.66, p=0.209). This was supported by the PI for clinical decision-making not including the null point, suggesting future studies will demonstrate positive effects, whereas the PI for survival rates included no effect. One study^64^ suggested feedback to EMS personnel may improve patient satisfaction, but was low quality and effect sizes did not reach statistical significance. The number of studies in the subgroup analyses (online supplemental appendix 5) was small and results not statistically significant.

The remaining six evaluative interventional studies were not included in the meta-analysis due to three not providing sufficient data to calculate effect sizes,^60 69 70^ two not including a comparison^71 72^ and one conducting qualitative analysis.^73^ The unstandardised effect sizes of the three studies with insufficient data indicated positive effects in the areas of protocol adherence, ambulance times and clinical decision-making (online supplemental appendix 6).^60 69 70^ The other three studies provided descriptive data on subjective self-reported measures, such as job satisfaction *(*‘*making clinical shifts more enjoyable*’,^71^ ‘*increased motivation*’^72^), team climate (‘*improved relationships with colleagues’*,^71 73^ ‘*increased organisational commitment*’^72^) and clinical decision-making (‘*avoid repeating experienced colleagues’ mistakes*’,^73^ ‘*improved confidence*’^71 73^).

### Mechanisms of feedback effects (interventional and non-interventional studies)

**Figure 3:**
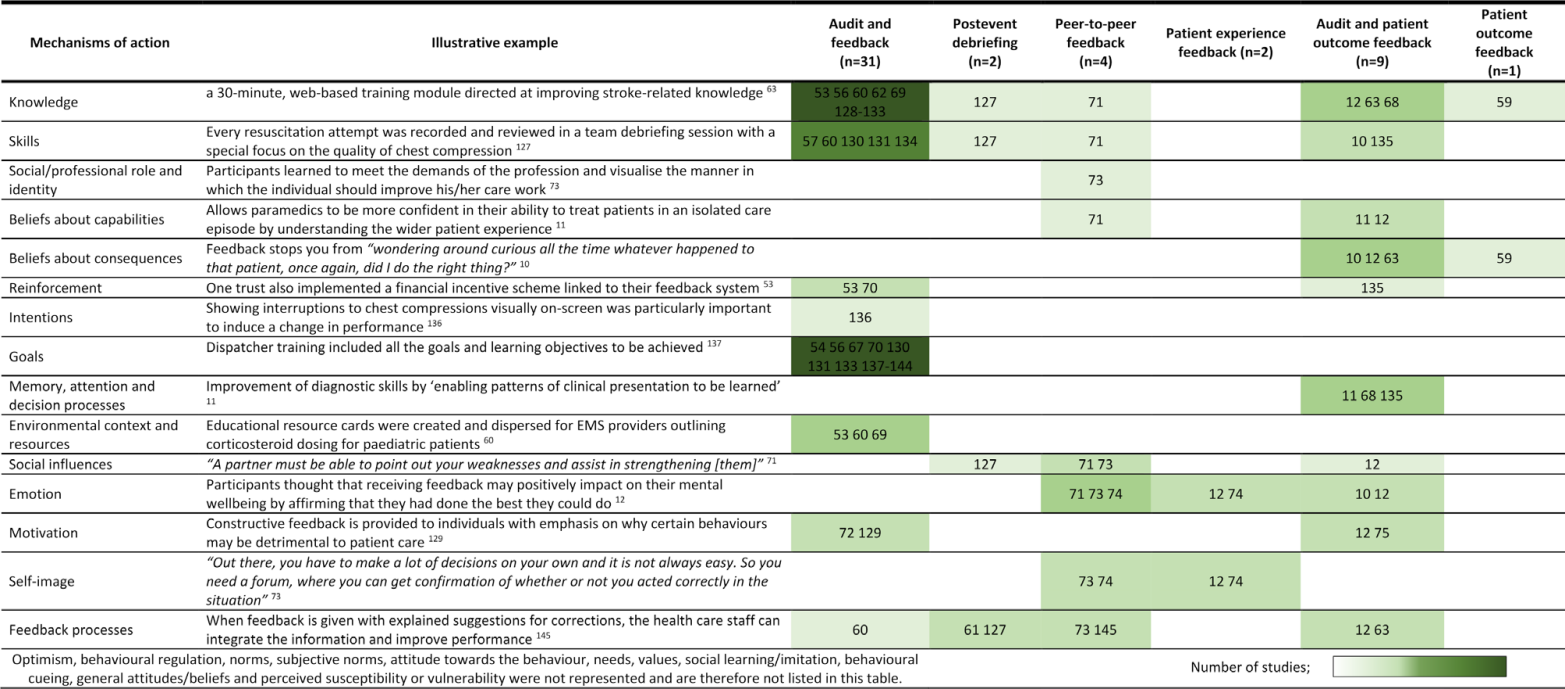
Potential mechanisms of feedback effects by feedback type.

Mechanisms of EMS feedback effects were poorly reported in interventional and non-interventional studies, with none exploring how mechanisms linked to study outcomes. Figure 3 presents deductive analysis of causal mechanisms for reported feedback effects based on coding to an established list of 26 mechanisms from behaviour change theory.^36^

Some mechanisms, such as knowledge, skills and feedback processes, were identified across different feedback types, while others, such as professional role and identity, environmental context and decision processes, were unique to specific feedback types. Audit and feedback interventions appeared to achieve their effects by influencing intentions and goals; while interventions, also including patient outcome information, appeared to include beliefs about consequences and decision processes. Meanwhile, postevent debriefing, peer-to-peer feedback and patient experience feedback interventions included causal mechanisms used by audit and patient outcome feedback, such as social influences and emotions. Without inferring causal mechanisms from limited information, no potential mechanisms could be identified for nine studies, including the incident-prompted feedback intervention.

### EMS professionals’ evaluation of feedback provision (non-interventional studies)

The non-interventional studies all reported that EMS professionals were dissatisfied with current feedback provision and desired more feedback, particularly patient outcome feedback.^10–12 31 74 75^ Concerns were that feedback was skewed towards the negative^10 75^ and only triggered by highly traumatic incidents,^11^ while EMS professionals desired routine, frequent and high-quality feedback.^10–12^ One UK study suggested that EMS professionals desired ‘pull’-type feedback, that is, initiated by individual clinicians, delivered electronically or involving staff mediators.^11^ In addition to the feedback types identified in interventional studies (table 2), non-interventional studies also discussed patient experience feedback, which involved EMS professionals receiving feedback from patients or relatives on the care they provided.

### Key contextual factors for feedback interventions (non-interventional studies)

The main barriers reported were practical, such as the lack of linkage between EMS and in-hospital information systems^68^ and the time and resources required for generating feedback.^75^ Potential social barriers were also highlighted, including how staff interact with and respond to feedback, especially within EMS culture where feedback may be perceived as linked to disciplinary action or being of questionable value.^10 12^ A reported ethical barrier was sharing of confidential patient details, especially for non-hospital-affiliated EMS agencies in the USA.^10 75^

Potential facilitators for effective feedback interventions were use of electronic health records to reduce demands of information capture and feedback delivery, thereby promoting sustainability.^11 76 77^ It was further reported that having clearly defined performance measures may support the provision of high-quality feedback.^68^ One study described a shift in EMS culture towards being more accepting of discussing mistakes, which may encourage clinicians to engage with feedback.^10^

## Discussion

The present review aimed to address a gap in existing evidence synthesis for feedback within EMS by summarising the literature on the types and effects of feedback received by EMS professionals. Previous reviews in the prehospital emergency care setting have focused on automated feedback from defibrillators^17–20^ and debriefing after simulation.^21–25^ Further to this, a non-systematic literature review from 2018 descriptively summarised a selection of the published literature relating to EMS clinical feedback.^78^ The present review sought to overcome previous reviews’ limitations by not restricting inclusion to randomised controlled trials, taking a broader view on feedback, drawing on explanatory theory and employing systematic search and evidence synthesis methods.

The reviewed interventional studies indicate that the source, content and mode of feedback interventions within EMS vary greatly, while design elements are poorly reported. Feedback interventions within EMS targeted and measured a number of quality and safety outcomes, including protocol adherence, patient satisfaction, survival rates, documentation, clinical decision-making, cardiac arrest performance and ambulance times. The meta-analyses of 30 evaluative interventional studies revealed substantial heterogeneity, so the moderate positive effect overall (d=0.50 (0.34, 0.67)) must be treated with caution, although it is comparable with recent systematic reviews within audit and feedback reporting (d=0.40).^5 47^ Our analysis indicated high heterogeneity for the majority of outcome categories, but found only moderate heterogeneity for clinical decision-making, which suggests existing evidence may more consistently support the positive point estimate in this outcome category. The high proportion of positive effects across individual studies and pooled effects for clinical decision-making suggests feedback to EMS personnel has a positive impact; however, the current evidence does not support a single point estimate of the aggregated effect of EMS feedback across multiple intervention types and outcomes. The high levels of clinical and statistical heterogeneity indicate a clear need to further examine different feedback types and identify which mechanisms are most effective within EMS.

Reviewed studies indicate that the current design of feedback interventions within EMS is for the large part atheoretical, which is a significant barrier to understanding feedback design and effectiveness. Analysis using behaviour change theory suggested there may be unique mechanisms for different feedback types, that is, audit and feedback (*intention/goals*), patient outcome feedback (*beliefs about consequences*) and peer-to-peer feedback (*professional role/self-image*), though we were unable to demonstrate a statistical difference between interventions due to small numbers of studies in each subgroup.

### Implications and recommendations

Interventions within EMS that explore patient outcome and patient experience feedback are needed as these were desired by staff in non-interventional studies yet under-represented in interventional studies. Considerable opportunity exists to enhance feedback provision to EMS personnel through data linkage and integrated data sets that span service boundaries.^76 77^ Similarly, enhancing existing electronic record systems might facilitate disaggregation of data to an individual level and enable targeted queries that support ‘pull model’ feedback, thereby making feedback more relevant to the recipient.^11 76^ As part of designing and implementing feedback interventions, information governance and ethical concerns linked to patient confidentiality should be addressed by bringing together relevant stakeholders.^11 12^

Beyond the literature included in this review, the proliferation of unpublished local feedback initiatives within EMS suggests that a review of current practice is needed to understand whether the published evidence is being implemented in current feedback provision. Poor reporting of intended mechanisms of action and a lack of theoretical underpinning indicate limited understanding of feedback mechanisms, consistent with the wider literature.^79 80^

The broader literature suggests specific opportunities for enhanced feedback to EMS personnel, such as combined audit and patient outcome feedback to improve decision-making^81–84^ for specific patient presentations (eg, cardiac arrest,^85–93^ myocardial infarction,^94–96^ stroke,^97 98^ abdominal pain,^99^ paediatrics,^100^ trauma^101 102^). Furthermore, patient outcome feedback may improve staff mental health and learning^103–109^ for patients not conveyed to hospital,^110^ with non-specific complaints,^111^ with significant differences between EMS and in-hospital diagnoses^112^ and patients referred to the coroner,^113^ while increased audit and feedback of particular skills (eg, intubation,^114^ ultrasound^115^) or situations (eg, handover,^116 117^ triage^118^) may improve performance. Lastly, increasing peer-to-peer and patient outcome feedback for certain staff groups may provide peer support and improve patient management skills for newly qualified^119 120^ and specialist paramedics.^121–126^

### Strengths and limitations

This is the first systematic review to investigate feedback within EMS, by adopting a broad definition of feedback and a rigorous methodology, which was published and registered a priori. The search strategy was developed from Cochrane reviews in audit and feedback, with the addition of subject matter expertise to increase sensitivity to the prehospital emergency care setting. The inter-rater reliability check indicated good reliability, with the lower kappa value for the intervention criterion potentially being explained by its more complex nature. Despite adopting a rigorous, systematic approach, the search strategy may not have identified every relevant article. Excluding articles without full-text English language translations may have removed potentially relevant studies and as with all systematic reviews, findings are subject to publication bias.

Although we were able to conduct a meta-analysis of a subset of quantitative evaluative studies (n=30, 83%), heterogeneity and variations in reporting quality limit the ability to recommend prioritisation of any specific feedback intervention or design feature. Our meta-analysis synthesised the available evidence, which did not include any randomised controlled trials and indicated high levels of heterogeneity; therefore, evidence for effects must be treated with caution despite using robust synthesis methods. Due to feedback interventions within EMS frequently being reported as part of multifaceted interventions with interacting components, it was not always possible to identify clearly whether a potential mechanism related to the feedback aspect of the intervention.

## Conclusions

In summary, this review demonstrated that the evidence base currently does not support a clear single point estimate of the pooled effect of feedback within EMS. Our meta-analysis revealed moderate positive effects but substantial heterogeneity across a range of quality and safety outcomes, including protocol adherence, patient satisfaction, survival rates, documentation, clinical decision-making, cardiac arrest performance and ambulance times. Viewed in the context of the existing audit and feedback literature, feedback within EMS is still in its infancy. Further research is needed to provide guidance and frameworks supporting better design and evaluation of feedback interventions within EMS in order to strengthen the evidence base. There is a clear need to develop a consensus about the key active components and mechanisms in feedback to EMS professionals.

## Data Availability

All data relevant to the study are included in the article or uploaded as supplementary information.
