# Supplementary material for: Types and effects of feedback for emergency ambulance staff: a systematic mixed studies review and meta-analysis
Source: BMJ Qual Saf. 2023 Apr 7;32(10):573–88. doi: 10.1136/bmjqs-2022-015634 (PMC10512001; doi:10.1136/bmjqs-2022-015634)
Supplement: Supplementary data [file bmjqs-2022-015634supp001.pdf]

Appendix 1: Search strategy for MEDLINE

Concept A: Ambulance Staff	Concept B: Feedback	Concept C: Feedback Content
(Ambulances or "Emergency Medical Technician" or "Air Ambulances" or "Emergency Medical Services" or Triage or Hotlines or "Call Centers" or "Emergency Medical Dispatch").sh. or (Paramedic* or EMS or Prehospital or Pre-hospital or "first responder*" or "emergency medical technician*" or "emergency service*" or Ambulance* or HEMS or "field triage" or "out-of-hospital" or 999 or 911 or 9-1-1 or dispatch* or EMD or "control cent*" or "call cent*" or "call handler*" or "call operator*" or "call?taker*" or "emergency operator*" or "telephone triage" or "emergency telecommunication" or TCPR or "emergency communication").tw. or (EMT* not (cancer or gene or tumo?r)).tw.	feedback.sh. or (feedback or post?box or debrief* or dashboard* or "clinical safety charts" or "extensive review" or "review sessions" or "follow?up tool" or "report* back or benchmark* or scorecard* or appraisal* or feedforward).tw.	(Quality Improvement or Quality of Health Care).sh. or ("clinical outcome*" or (chang* adj3 behavio?r) or performance or "quality of care" or conveyance or "quality improvement*" or “service improvement*” or "professional development" or "patient outcome*" or diagnos?s or (quality adj3 ("chest compression*" or CPR or "cardio?pulmonary resuscitation" or ALS)) or "treatment time*" or "coroners report*" or (adher* adj2 (system* or guideline*)) or "quality data" or decision?making or "patient safety" or well?being or reflection).tw.

Appendix 2: Data extraction template

OVERALL CATEGORY	SPECIFIC CATEGORY	OPTIONS (IF APPLICABLE)	
Reference information	Author		
	Title		
	Year		
	Journal		
Study overview	Study country		
	Study category	1 – Interventional feedback study	
		2 – Non-interventional feedback study	
		3 – Other study with feedback element	
	Context	*e.g. paramedic emergency services, emergency operations centre*	
	Study MMAT category & study design	Qualitative	Ethnography
			Phenomenology
			Narrative research
			Grounded theory
			Case study
			Qualitative description
		Quantitative RCT	RCT
		Quantitative non randomised	Non-RCT
			Cohort study
			Case-control study
			Cross-sectional analytic study
			Time series
		Quantitative descriptive	Incidence/prevalence study without comparison
			Survey
			Case series
			Case report
		Mixed methods	Convergent design
			Sequential explanatory design
			Sequential exploratory design
	Study design as defined by the authors		
	Study purpose		
	Study's definition of feedback		
	Study findings		
	Suggested further study		
Study	Number		

participants	Professional background	
	Reported demographics	
Study outcome measures (+ corresponding results)	Staff wellbeing	
	Quality + safety of patient care	
	Professional development	
	Clinical decision-making	
	Other clinically relevant outcomes	
Interventional study characteristics	Brief name	
	Patient condition studied	
	Targeted behaviour	
	Direction of change required	Increase current behaviour/Decrease current behaviour/Change behaviour or mix/Unclear
	Baseline performance	Above average/Average/Below average/Not reported
	Extent to which the intervention was delivered as planned	
	Was there a significant positive effect on the primary outcome measure?	
	Source	Hospital/Ambulance service managers/Peers/Patients
	Content	
	Mode	
	Format	
	Visual or graphical elements	
	Frequency	
	Duration of intervention	
	Lag-time	
	Time/resources involved in generating feedback	
	Study length	
	Recipient level	Individual/Group/Individual+group
	Patient cases	Individual/Aggregate/Individual+aggregate
	Feedback alone or multifaceted intervention	Feedback alone<br>Feedback + reminders<br>Feedback + educational outreach<br>Feedback + educational intervention<br>Feedback + organisational interventions<br>Feedback + financial incentives<br>Feedback + patient-mediated interventions
	Push or pull model?	Push model/Pull model/Unclear
	Recipient participation	Yes/No/Unclear
	Message framing	
	Comparator	
	Instructions for improvement	Explicit, measurable target/Action

		plan/Both/Neither
	Action plans accompanying the feedback	
	Underlying theory	
	Feedback categories	Audit & feedback/Post-event debriefing/Peer-to-peer feedback/Incident prompted feedback/Audit & patient outcome feedback/Patient outcome feedback
Non-interventional study characteristics	Current provision	
	Feedback content	
	Motives for seeking feedback	
	Mechanisms for feedback	
	Barriers	
	Moderators	
	Antecedents: Feedback recipient	
	Antecedents: Context	
Mixed methods appraisal tool	Are there clear research questions?	Yes/No/Can't tell
	Do the collected data allow to address the research questions?	Yes/No/Can't tell
Qualitative	Is the qualitative approach appropriate to answer the research question?	Yes/No/Can't tell
	Are the qualitative data collection methods adequate to address the research question?	Yes/No/Can't tell
	Are the findings adequately derived from the data?	Yes/No/Can't tell
	Is the interpretation of results sufficiently substantiated by data?	Yes/No/Can't tell
	Is there coherence between qualitative data sources, collection, analysis and interpretation?	Yes/No/Can't tell
Quantitative RCTs	Is randomization appropriately performed?	Yes/No/Can't tell
	Are the groups comparable at baseline?	Yes/No/Can't tell
	Are there complete outcome data?	Yes/No/Can't tell
	Are outcome assessors blinded to the intervention provided?	Yes/No/Can't tell
	Did the participants adhere to the assigned intervention?	Yes/No/Can't tell
Quantitative non-randomised	Are the participants representative of the target population?	Yes/No/Can't tell
	Are measurements appropriate regarding both the outcome and intervention (or exposure)?	Yes/No/Can't tell

	Are there complete outcome data?2	Yes/No/Can't tell
	Are the confounders accounted for in the design and analysis?	Yes/No/Can't tell
	During the study period, is the intervention administered (or exposure occurred) as intended?	Yes/No/Can't tell
Quantitative descriptive	Is the sampling strategy relevant to address the research question?	Yes/No/Can't tell
	Is the sample representative of the target population?	Yes/No/Can't tell
	Are the measurements appropriate?	Yes/No/Can't tell
	Is the risk of nonresponse bias low?	Yes/No/Can't tell
	Is the statistical analysis appropriate to answer the research question?	Yes/No/Can't tell
Mixed methods	Is there an adequate rationale for using a mixed methods design to address the research question?	Yes/No/Can't tell
	Are the different components of the study effectively integrated to answer the research question?	Yes/No/Can't tell
	Are the outputs of the integration of qualitative and quantitative components adequately interpreted?	Yes/No/Can't tell
	Are divergences and inconsistencies between quantitative and qualitative results adequately addressed?	Yes/No/Can't tell
	Do the different components of the study adhere to the quality criteria of each tradition of the methods involved?	Yes/No/Can't tell

Appendix 3: Details of included studies

Interventional feedback studies within EMS – Evaluative studies (n=36)

Author	Year	Country	Context	MMAT category	Study design	Quality assessment	Number of participants	Professional background	Outcome measures/findings
**Bahouth**	2022	Israel	Paramedic Emergency Services	Quantitative non randomised	Cohort study	Low-moderate	518	Emergency Medical Technicians + Paramedics	• Time in field<br>• Field file not present<br>• Neck collar fixation not performed
**Bleijenberg**	2017	Netherlands	Paramedic Emergency Services	Quantitative non randomised	Cross-sectional study	High-moderate	124	Patients	• Number of delivered chest compressions in one minute
**Bobrow2**	2016	USA	Emergency Operations Centre	Quantitative non randomised	Cross-sectional study	High	2334	Patients	• Provision of telephone cardiopulmonary resuscitation<br>• Survival to hospital discharge
**Brink**	2012	Sweden	Paramedic Emergency Services	Qualitative	Qualitative descriptive study	High	10	Emergency Medical Technicians + Paramedics	• Improved relationships with colleagues<br>• Avoid repeating experienced colleagues' mistakes<br>• Improved confidence
**Choi**	2014	USA	Paramedic Emergency Services	Quantitative non randomised	Cross-sectional study	Low-moderate	1176	Patients	• Percentage of last known well time documented<br>• Percentage of prenotification given
**Clawson**	1998	USA	Emergency Operations Centre	Quantitative non randomised	Cross-sectional study	Moderate	32	Emergency operations centre staff	• Mean overall compliance score
**Daudelin**	2012	USA	Paramedic Emergency Services	Quantitative non randomised	Cross-sectional study	High	6994	Patients	• Performance of a prehospital electrocardiogram<br>• EMS run time
**DelliFraine**	2013	USA	Ambulance Service - Organisatio	Quantitative non randomised	Cohort study	High	24	EMS organisations	• Median symptom to balloon time

			nal Level						
**Ebbs**	2012	Australia	Paramedic Emergency Services	Quantitative non randomised	Cross-sectional study	Low-moderate	227	Emergency Medical Technician + Paramedic	• Key performance indicator results
**Eckstein**	1999	USA	Paramedic Emergency Services	Quantitative non randomised	Cross-sectional study	Moderate	7103	Patients	• Mortality rates amongst fallouts<br>• Fallout rate of penetrating trauma patients with on scene times >20 minutes
**Gevers**	2020	South Africa	Paramedic Emergency Services	Quantitative descriptive	Survey	Moderate	50	EMS personnel	• Improved confidence<br>• Making clinical shifts more enjoyable<br>• Improved relationships with colleagues
**Gropen**	2019	USA	Emergency Operations Centre	Quantitative non randomised	Cohort study	Moderate	24	Paramedics/communicators	• Ability of EMS providers to predict large vessel occlusion
**Hardeland**	2017	Norway	Emergency Operations Centre	Quantitative non randomised	Cross-sectional study	Moderate	561	Patients	• Immediate recognition of cardiac arrest<br>• Ambulance response interval
**Hermans**	2017	Netherlands	Ambulance Service Organisation	Quantitative non randomised	Cross-sectional study	High-moderate	441	Patients	• First medical contact to balloon time
**Hopkins**	2016	USA	Paramedic Emergency Services	Quantitative non randomised	Cross-sectional study	High-moderate	737	Patients	• Neurologically intact survivors
**Hubner**	2017	Austria	Paramedic Emergency Services	Quantitative non randomised	Cross-sectional study	High-moderate	2209	Patient	• Time to first medical contact<br>• Hands-off interval longer than 30 seconds<br>• Survival to hospital discharge
**Joyce**	1997	USA	Paramedic Emergency Services	Quantitative non randomised	Cross-sectional study	Moderate	1862	Emergency Medical Technician +	• Response time<br>• Adequate documentation pertaining to physical exam

								Paramedic	• Protocol followed or deviation justified<br>• Release at scene appropriate
**Lukas**	2012	Germany	Paramedic Emergency Services	Quantitative non randomised	Case-control study	High-moderate	295	Paramedics & emergency physicians on ambulances	• Observed return of spontaneous circulation
**Lyon**	2012	UK	Paramedic Emergency Services	Quantitative non randomised	Cross-sectional study	Moderate	111	Cardiac arrest traces	• Median time-to-shock interval
**Niles**	2010	USA	Paramedic Emergency Services	Quantitative non randomised	Cross-sectional study	Moderate	178	Patients	• Percentage of patients who had a prehospital electrocardiogram performed<br>• False Primary Percutaneous Coronary Intervention activations by EMS
**Noble**	2020	Rwanda	Paramedic Emergency Services	Mixed methods	Sequential explanatory design	High	34	Ambulance drivers, anaesthetists + nurses	• Increased motivation
**O'Connor**	1994	USA	Paramedic Emergency Services	Quantitative non randomised	Cross-sectional study	Moderate	14000	Prehospital reports/patients	• Endotracheal tube success rate<br>• Endotracheal tube missing documentation rate<br>• Trauma scene times <10 minutes
**Olasveengen**	2007	England, Sweden, Norway	Paramedic Emergency Services	Quantitative non randomised	Cross-sectional study	Moderate	124	Patients	• Chest compressions per minute
**Oostema**	2019	USA	Paramedic Emergency Services	Quantitative non randomised	Cross-sectional study	High-moderate	1805	Patients	• Stroke recognition rate<br>• Hospital prenotification<br>• Cincinnati Prehospital Stroke Scale documentation rates<br>• Scene time <15 minutes
**Park**	2018	South	Emergency	Quantitative	Cross-	High-	12670	Patients	• Prehospital return of

		Korea	Operations Centre + Paramedic Emergency Services	non randomised	sectional study	moderate			spontaneous circulation
**Persse**	2002	USA	Paramedic Emergency Services	Quantitative non randomised	Cohort study	Low-moderate	151	Patients	• Non-transport decision made by paramedics<br>• Satisfaction level of patients
**Riney**	2021	USA	Paramedic Emergency Services	Quantitative non randomised	Cross-sectional study	Moderate	256	Patient encounters	• Proportion of children receiving prehospital corticosteroids for asthma exacerbation
**Scholz1**	2008	German y	Paramedic Emergency Services	Quantitative non randomised	Cross-sectional study	Moderate	147	Patients	• Contact-to-balloon time<br>• Patients transported directly to the catheterisation laboratory
**Scholz2**	2012	German y	Paramedic Emergency Services	Quantitative non randomised	Cross-sectional study	Moderate	1183	Patients	• Proportion of contact-to-balloon time < 120 minutes<br>• Patients transported directly to the catheterisation laboratory<br>• 1-year mortality of patients
**Scholz3**	2020	German y	Paramedic Emergency Services	Quantitative non randomised	Cross-sectional study	Moderate	13219	Patients	• Percentage of patients with prehospital electrocardiogram recordings<br>• Contact-to-balloon time < 90 minutes<br>• In-hospital mortality
**Scott**	2017	Rwanda	Paramedic Emergency Services	Quantitative non randomised	Cross-sectional study	High-moderate	3822	Patients	• Supplemental oxygen administration for hypoxia
**Siriwardena**	2014	UK	Paramedic Emergency Services	Mixed methods	Converge nt design	High	12	Ambulance services	• Stroke care bundle delivery
**Swor**	1990	USA	Paramedic Emergency Services	Quantitative non randomised	Cross-sectional study	Moderate	100	Paramedics	• Number of deviations from protocol

**Tanaka**	2012	Japan	Emergency Operations Centre	Quantitative non randomised	Cross-sectional study	High-moderate	4995	Patients	• Incidence of telephone cardiopulmonary resuscitation<br>• Survival with favourable neurological outcomes
**Todt**	2013	Sweden	Paramedic Emergency Services	Quantitative non randomised	Cross-sectional study	Low-moderate	156	Patients	• Time from electrocardiogram to decision for Primary Percutaneous Coronary Intervention
**Weston**	2018	USA	Paramedic Emergency Services	Quantitative non randomised	Cross-sectional study	High-moderate	964	Patients	• Percentage of episodes that met goal compression depth >5 cm greater than 90% of the time

Interventional feedback studies within EMS – Descriptive case studies (n=6)

Author	Year	Country	Context	MMAT category	Study design	Quality assessment	Number of participants	Professional background
**Clarke**	2014	UK	Paramedic Emergency Services	Quantitative non randomised	Cross-sectional study	Low-moderate	8	Paramedic
**Lindstrom**	2011	Sweden	Emergency Operations Centre	Quantitative descriptive	Incidence/prevalence study without comparison	High	530	Patients/assignments
**Scholz4**	2020	Germany	Paramedic Emergency Services	Quantitative non randomised	Cross-sectional study	Moderate	4926	Patients
**Scholz5**	2021	Germany	Paramedic Emergency Services	Quantitative non randomised	Cross-sectional study	Moderate	20005	Patients
**Stella**	2010	Australia	Paramedic Emergency Services	Quantitative descriptive	Incidence/prevalence study without comparison	Moderate	454	Patient encounters
**Walters**	1992	UK	Paramedic Emergency	Quantitative descriptive	Incidence/prevalence study without comparison	High-moderate	190	Ambulance attendants

			Services					

Non-interventional feedback studies within EMS (n=6)

Author	Year	Country	Context	MMAT category	Study design	Quality assessment	Number of participants	Professional background
**Cash**	2017	USA	Paramedic Emergency Services	Quantitative descriptive	Survey	High-moderate	15766	Emergency Medical Technician + Paramedic
**Eaton-Williams**	2020	UK	Paramedic Emergency Services	Qualitative	Phenomenology	High	8	Emergency Medical Technician + Paramedic
**McGuire**	2021	USA	Paramedic Emergency Services	Quantitative descriptive	Survey	Moderate	94	Ambulance staff
**Mock**	1997	USA	Paramedic Emergency Services	Quantitative descriptive	Incidence/prevalence study without comparison	High	69	Emergency Medical Technician + Paramedic
**Morrison**	2017	Canada	Paramedic Emergency Services	Qualitative	Interpretive descriptive analysis	High	12	Paramedic
**Wilson**	2022	UK	Paramedic Emergency Services	Qualitative	Thematic analysis	High	24	Emergency Medical Technician + Paramedic

Appendix 4: Quality assessment

Qualitative Studies	**Are there clear research questions?**	**Do the collected data allow to address the research questions?**	Is the qualitative approach appropriate to answer the research question?	Are the qualitative data collection methods adequate to address the research question?	Are the findings adequately derived from the data?	Is the interpretation of results sufficiently substantiated by data?	Is there coherence between qualitative data sources, collection, analysis and interpretation?
**Brink**	Yes	Yes	Yes	Yes	Yes	Yes	Yes
**Eaton-Williams**	Yes	Yes	Yes	Yes	Yes	Yes	Yes
**Morrison**	Yes	Yes	Yes	Yes	Yes	Yes	Yes
**Wilson**	Yes	Yes	Yes	Yes	Yes	Yes	Yes

Quantitative non randomised studies	**Are there clear research questions?**	**Do the collected data allow to address the research questions?**	Are the participants representative of the target population?	Are measurements appropriate regarding both the outcome and intervention?	Are there complete outcome data?	Are the confounders accounted for in the design and analysis?	During the study periods, is the intervention administered as intended?
**Bahouth**	Yes	Yes	Can't tell	Yes	Yes	No	Can’t tell
**Bleijenberg**	Yes	Yes	Yes	Yes	Yes	No	Yes
**Bobrow2**	Yes	Yes	Yes	Yes	Yes	Yes	Yes
**Choi**	Yes	Yes	Can't tell	Yes	Yes	No	Can't tell
**Clarke**	Yes	Yes	Can't tell	No	Yes	No	Yes
**Clawson**	Yes	Yes	Yes	Yes	Yes	No	Can't tell
**Daudelin**	Yes	Yes	Yes	Yes	Yes	Yes	Yes
**DelliFraine**	Yes	Yes	Yes	Yes	Yes	Yes	Yes
**Ebbs**	Yes	Yes	Can't tell	Yes	No	No	Can't tell
**Eckstein**	Yes	Yes	Yes	Yes	Yes	No	Can't tell
**Gropen**	Yes	Yes	Yes	Yes	Yes	No	Can't tell
**Hardeland**	Yes	Yes	Yes	Yes	Yes	No	Can't tell
**Hermans**	Yes	Yes	Yes	Yes	Yes	Yes	Can't tell
**Hopkins**	Yes	Yes	Yes	Yes	Yes	Yes	Can't tell
**Hubner**	Yes	Yes	Yes	Yes	Yes	Yes	Can't tell
**Joyce**	Yes	Yes	Yes	Yes	Yes	No	Can't tell
**Lukas**	Yes	Yes	Yes	Yes	Yes	Yes	Can't tell
**Lyon**	Yes	Yes	Yes	Yes	Yes	No	Can't tell
**Niles**	Yes	Yes	Yes	Yes	Yes	No	Can't tell
**O'Connor**	Yes	Yes	Yes	Yes	Yes	No	Can't tell
**Olasveengen**	Yes	Yes	Yes	Yes	Yes	No	Can't tell
**Oostema**	Yes	Yes	Yes	Yes	Yes	Yes	Can't tell
**Park**	Yes	Yes	Yes	Yes	Yes	Yes	Can't tell
**Persse**	Yes	Yes	Can't tell	Yes	Yes	No	Can't tell
**Riney**	Yes	Yes	Yes	Yes	Yes	No	Can't tell

**Scholz1**	Yes	Yes	Yes	Yes	Yes	No	Can't tell
**Scholz2**	Yes	Yes	Yes	Yes	Yes	No	Can't tell
**Scholz3**	Yes	Yes	Yes	Yes	Yes	No	Can't tell
**Scholz4**	Yes	Yes	Yes	Yes	Yes	No	Can't tell
**Scholz5**	Yes	Yes	Yes	Yes	Yes	No	Can't tell
**Scott**	Yes	Yes	Yes	Yes	Yes	Yes	Can't tell
**Swor**	Yes	Yes	Yes	Yes	Yes	No	Can't tell
**Tanaka**	Yes	Yes	Yes	Yes	Yes	Yes	Can't tell
**Todt**	Yes	Yes	Can't tell	Yes	Yes	No	Can't tell
**Weston**	Yes	Yes	Yes	Yes	Yes	Yes	Can't tell

Quantitative descriptive studies	**Are there clear research questions?**	**Do the collected data allow to address the research questions?**	Is the sampling strategy relevant to address the research question?	Is the sample representative of the target population?	Are the measurements appropriate?	Is the risk of nonresponse bias low?	Is the statistical analysis appropriate to answer the research question?
**Cash**	Yes	Yes	Yes	Can't tell	Yes	Can't tell	Yes
**Gevers**	Yes	Yes	Yes	Can't tell	Yes	Can't tell	Yes
**Lindstrom**	Yes	Yes	Yes	Yes	Yes	Yes	Yes
**McGuire**	Yes	Yes	Yes	Can't tell	Yes	Can't tell	Yes
**Mock**	Yes	Yes	Yes	Yes	Yes	Yes	Yes
**Stella**	Yes	Yes	Yes	Can't tell	Yes	Can't tell	Yes
**Walters**	Yes	Yes	Yes	Yes	Yes	Can't tell	Yes

Mixed methods studies	**Are there clear research questions?**	**Do the collected data allow to address the research questions?**	Is there an adequate rationale for using a mixed methods design to address the research question?	Are the different components of the study effectively integrated to answer the research question?	Are the outputs of the integration of qualitative and quantitative components adequately interpreted?	Are divergences and inconsistencies between quantitative and qualitative results adequately addressed?	Do the different components of the study adhere to the qualitative criteria of each tradition of the methods involved?
**Noble**	Yes	Yes	Yes	Yes	Yes	Yes	Yes
**Siriwardena**	Yes	Yes	Yes	Yes	Yes	Yes	Yes

Appendix 5: Subgroup analyses by feedback type, feedback source, recipient level, feedback alone, aggregation level and format

	d	95% CI	p	I^2	95% CI	$p_{Subgroup}$
**Study quality**						**0.517**
▪ High	0.24	-0.27-0.76	0.352	0.99	0.98-1.00	
▪ High-moderate	0.46	-0.64-1.57	0.452	0.98	0.89-0.99	
▪ Moderate	0.63	-0.46-1.72	0.180	0.97	0.92-0.98	
▪ Low-moderate	0.41	-0.77-1.59	0.619	0.97	0.92-0.99	
**Feedback type**						**0.405**
▪ Audit & feedback	0.56	0.37-0.75	<0.001*	0.99	0.97-0.99	
▪ Feedback combined	0.43	-0.23-1.09	0.592	0.98	0.95-1.00	
▪ Peer-to-peer feedback	0.01	-0.91-0.92	0.135	0.98	0.93-1.00	
▪ Post-event debriefing	0.10	-1.25-1.45	0.429	-	-	
**Feedback source**						**0.787**
▪ Hospital	0.63	-2.00-3.26	0.798	0.97	0.85-1.00	
▪ Researchers	0.47	-1.93-2.87	0.572	0.98	0.89-0.99	
▪ Electronic dashboard	0.81	-0.37-2.00	0.172	-	-	
▪ Ambulance service managers	0.61	-1.80-3.01	0.731	0.99	0.92-0.99	
▪ Medical director	0.61	-1.76-3.31	0.774	0.97	0.92-1.00	
▪ Peers	0.01	-2.56-2.58	0.249	0.98	0.93-1.00	
▪ Unclear	0.40	-2.03-2.84	0.515	0.96	0.90-0.99	
**Recipient level**						**0.535**
▪ Individual	0.71	0.35-1.06	<0.001*	0.99	0.92-1.00	
▪ Individual + organisation	0.09	-0.90-1.09	0.061	0.97	0.85-1.00	
▪ Individual + team	0.37	-0.55-1.30	0.250	0.98	0.95-1.00	
▪ Organisation	0.54	-0.35-1.43	0.542	0.99	0.98-1.00	
▪ Team	0.50	-0.32-1.31	0.365	0.88	0.69-0.95	
▪ Unclear	0.62	-0.58-1.81	0.836	0.32	0.00-0.96	
**Feedback alone**						**0.379**
▪ Feedback alone	0.58	-0.09-1.26	0.902	0.99	0.95-0.99	
▪ Feedback + educational intervention	0.56	0.27-0.84	<0.001*	0.99	0.95-0.99	
▪ Feedback + organisational intervention	0.31	-0.41-1.03	0.251	0.98	0.96-0.99	
**Level of aggregation**						**0.381**
▪ Individual	0.51	-0.35-1.12	0.341	0.97	0.91-0.98	
▪ Aggregate	0.73	0.35-1.12	<0.001*	0.99	0.98-1.00	
▪ Individual + aggregate	0.43	-0.44-1.31	0.221	0.96	0.92-0.98	
▪ Unclear	0.16	-0.92-1.24	0.101	0.38	0.00-0.99	
**Format**						**0.703**
▪ Verbal	0.55	-0.44-1.55	0.508	0.89	0.72-0.96	
▪ Written	0.47	-0.46-1.39	0.681	0.98	0.96-0.99	
▪ Verbal + written	0.72	-0.36-1.81	0.273	1.00	0.98-1.00	
▪ Unclear	0.37	-0.07-0.80	0.097	0.94	0.00-0.90	

Appendix 6: Unstandardized effect sizes for remaining evaluative interventional studies of feedback within EMS

Author and year	Outcome category	Outcome measure	Effect direction	Unstandardized effect size
Riney 2021	Protocol adherence	Proportion receiving systematic prehospital corticosteroids	Positive	Centreline shifted from 0% to 34%
Gropen 2019	Clinical decision-making	Ability of EMS providers to predict large vessel occlusion	Positive	Area under the curve 0.61 (95% CI: .44-.77) to 0.74 (95% CI: .64-.84)
DelliFraine 2013	Ambulance times	Symptom to balloon time	Positive	Median 195min to 162min, p<0.001
